# Carnosic Acid Inhibits the Epithelial-Mesenchymal Transition in B16F10 Melanoma Cells: A Possible Mechanism for the Inhibition of Cell Migration

**DOI:** 10.3390/ijms150712698

**Published:** 2014-07-17

**Authors:** So Young Park, Hyerim Song, Mi-Kyung Sung, Young-Hee Kang, Ki Won Lee, Jung Han Yoon Park

**Affiliations:** 1 Advanced Institutes of Convergence Technology, Seoul National University, Suwon, Gyonggi-do 443-270, Korea; E-Mail: young0122@hallym.ac.kr; 2 Department of Food Science and Nutrition, Hallym University, Chuncheon 200-702, Korea; E-Mails: hyerim0715@hallym.ac.kr (H.S.); yhkang@hallym.ac.kr (Y.-H.K.); 3 Department of Food and Nutrition, Sookmyung Women’s University, Seoul 140-742, Korea; E-Mail: mksung@sookmyung.ac.kr; 4 WCU Biomodulation Major, Department of Agricultural Biotechnology and Center for Food and Bioconvergence, Seoul National University, Seoul 151-921, Korea

**Keywords:** carnosic acid, melanoma, migration, epithelial-mesenchymal transition

## Abstract

Carnosic acid is a natural benzenediol abietane diterpene found in rosemary and exhibits anti-inflammatory, antioxidant, and anti-carcinogenic activities. In this study, we evaluated the effects of carnosic acid on the metastatic characteristics of B16F10 melanoma cells. When B16F10 cells were cultured in an *in vitro* Transwell system, carnosic acid inhibited cell migration in a dose-dependent manner. Carnosic acid suppressed the adhesion of B16F10 cells, as well as the secretion of matrix metalloproteinase (MMP)-9, tissue inhibitor of metalloproteinase (TIMP)-1, urokinase plasminogen activator (uPA), and vascular cell adhesion molecule (VCAM)-1. Interestingly, secretion of TIMP-2 increased significantly in B16F10 cells treated with 10 μmol/L carnosic acid. Additionally, carnosic acid suppressed the mesenchymal markers snail, slug, vimentin, and *N*-cadherin and induced epithelial marker E-cadherin. Furthermore, carnosic acid suppressed phosphorylation of Src, FAK, and AKT. These results indicate that inhibition of the epithelial-mesenchymal transition may be important for the carnosic acid-induced inhibition of B16F10 cell migration.

## 1. Introduction

The incidence of melanoma has been rising worldwide over the past two decades [1]. Melanoma is the fifth most frequent cancer in men and the seventh most recurrent cancer in women in the United States [2]. The majority of patients with primary melanomas can be successfully treated by surgical resection when the disease is diagnosed in a timely manner. However, metastatic melanoma constitutes more than 80% of deaths from skin cancer due to its aggressiveness and resistance to currently available therapies. Therefore, control of metastasis is very important in the management of melanoma [1,3].

Metastasis is a multifaceted process by which cancer cells spread from the original site and form tumors at distant sites. When invasive melanoma cells metastasize, their cytoskeleton, the extracellular matrix (ECM), and surrounding stromal cells reorganize [4]. Matrix metalloproteinases (MMPs), a family of zinc-dependent endopeptidases, and urokinse plasminogen activator (uPA) play a critical role in ECM degradation [5].

Tumor cells derived from epithelial tissues undergo changes in cell morphology including loss of cell polarity to metastasize to other parts of the body [6]. The changes in cell adhesion and migration during tumor invasion are analogous to an important developmental process termed the epithelial-mesenchymal transition (EMT) [7]. The EMT plays an important role in the progression and metastasis of cancer cells. Performed by a wide variety of epithelial carcinomas, the EMT has been proposed to endow cancer cells with increased motility and invasiveness during tumor dissemination [8]. Three key changes occur to cells during the EMT: epithelial cells gain mesenchymal markers (vimentin and fibronectin); they undergo major changes in their cytoskeleton allowing them to obtain mesenchymal phenotypes with increased invasiveness and motility; and they give up cell polarity, cell-to-cell contact, and epithelial markers, particularly E-cadherin [9].

Much effort has gone into searching for natural compounds with anti-cancer activities. Carnosic acid is one of the main active phenolic diterpenes present in rosemary that has been universally utilized as an important antioxidant in the food and cosmetic industries [10]. Carnosic acid exhibits anti-inflammatory activities [11]. Additionally, carnosic acid inhibits growth of several cancer cell types, including estrogen receptor (ER)-negative breast cancer cells [12] and human HL-60 and U937 leukemia cells [13]. Furthermore, oral administration of carnosic acid at 10 mg/kg body weight effectively prevents DMBA-induced tumor formation in the buccal pouch of golden Syrian hamsters [14]. Carnosic acid at 0–94 μmol/L inhibits migration, invasion, and viability of Caco2 human colon adenocarcinoma cells [15]. In the present study, we evaluated the effects of the carnosic acid on the metastatic characteristics of B16F10 melanoma cells. We demonstrate that carnosic acid potently inhibited the EMT and migration and adhesion of B16F10 cells at concentrations (2.5–10 μmol/L) that did not affect B16F10 cell viability.

## 2. Results

### 2.1. Carnosic Acid Inhibits Migration of B16F10 Melanoma Cells

**Figure 1:**
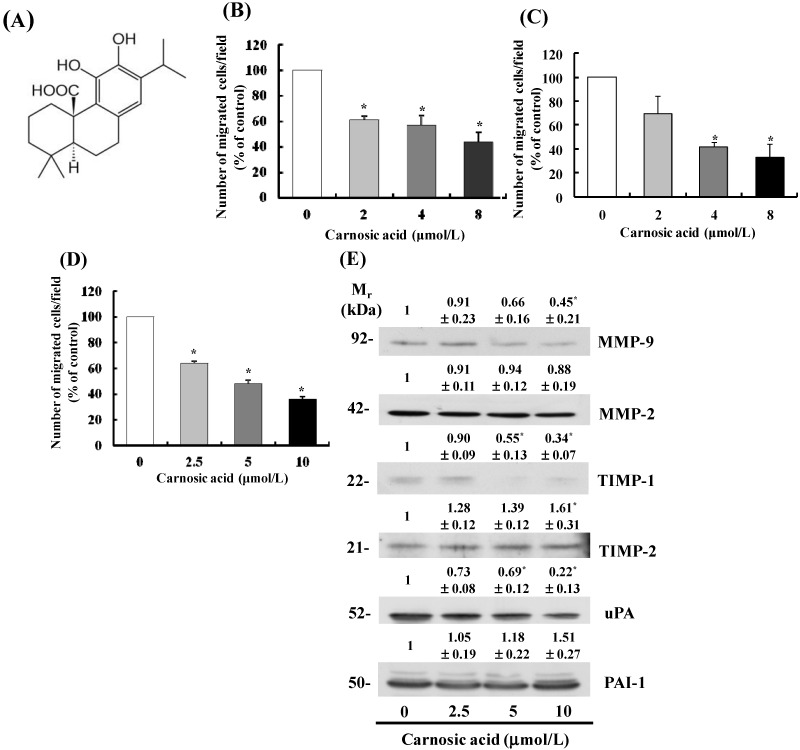
Carnosic acid inhibits migration of B16F10 melanoma cells. (**A**) Structure of carnosic acid; (**B**–**D**) Transwell migration assays were conducted with Lewis lung cancer (LLC) cells (2.5 × 10^4^ cells/well) (**B**); CT26 cells (2.5 × 10^4^ cells/well) (**C**); and B16F10 cells (5 × 10^4^ cells/well) (**D**) Migrating cells were quantified, and each bar represents the mean ± SEM from three independent experiments; (**E**) B16F10 cells were plated in 100 mm dishes at 1 × 10^6^ cells/dish in MEM supplemented with 10% FBS and 292 mg/L l-glutamine. After 12 h of incubation, the monolayers were serum-starved in MEM then treated for 12 h with carnosic acid. Conditioned media were collected and concentrated for Western blotting. The volumes of media loaded onto the gel were adjusted for equivalent proteins. Photographs of chemiluminescent detection of the immunoblots, which are representative of three independent experiments, are shown. Relative abundance of each band was estimated by densitometric scanning of the exposed films. The adjusted mean ± SEM (*n* = 3) of each band is shown above each blot. * Significantly different from the control (0 μmol/L carnosic acid), *p* < 0.05.

Transwell migration assays were performed to evaluate the effect of carnosic acid on metastatic activity. As shown in Figure 1D, carnosic acid (2.5–10 μmol/L) dose-dependently inhibited migration of B16F10 cells. Additionally, carnosic acid significantly inhibited the migration of Lewis lung cancer cells (LLC) (Figure 1B) and CT26 colon cancer cells (Figure 1C). However, the same concentrations of carnosic acid did not decrease cell viability when the cells were treated for 12 h with carnosic acid (data not shown); therefore, we utilized 2.5–10 μmol/L carnosic acid in subsequent B16F10 cell experiments.

### 2.2. Carnosic Acid Alters Secretion of MMPs, Tissue Inhibitor of Metalloproteinases (TIMPs), and uPA in B16F10 Cells

Degradation of the ECM by cancer cells is an important process in metastasis. It is catalyzed by various proteolytic enzymes that are produced and secreted by cancer cells [16]. Representative proteins that are involved in degradation of the ECM are MMPs, TIMPs, and uPA [17,18]. As carnosic acid significantly inhibited the migration of B16F10 cells, we next evaluated the effects of carnosic acid on secretion of these proteins by conducting Western blot analyses with conditioned media. The results demonstrated that secretion of MMP-9, TIMP-1, and uPA decreased in B16F10 cells treated with carnosic acid, whereas the level of TIMP-2 increased significantly in cells treated with 10 μmol/L carnosic acid. Secretion of MMP-2 and plasminogen activator inhibitor-1 (PAI-1) did not change significantly (Figure 1E).

### 2.3. Carnosic Acid Inhibits B16F10 Cell Adhesion

**Figure 2:**
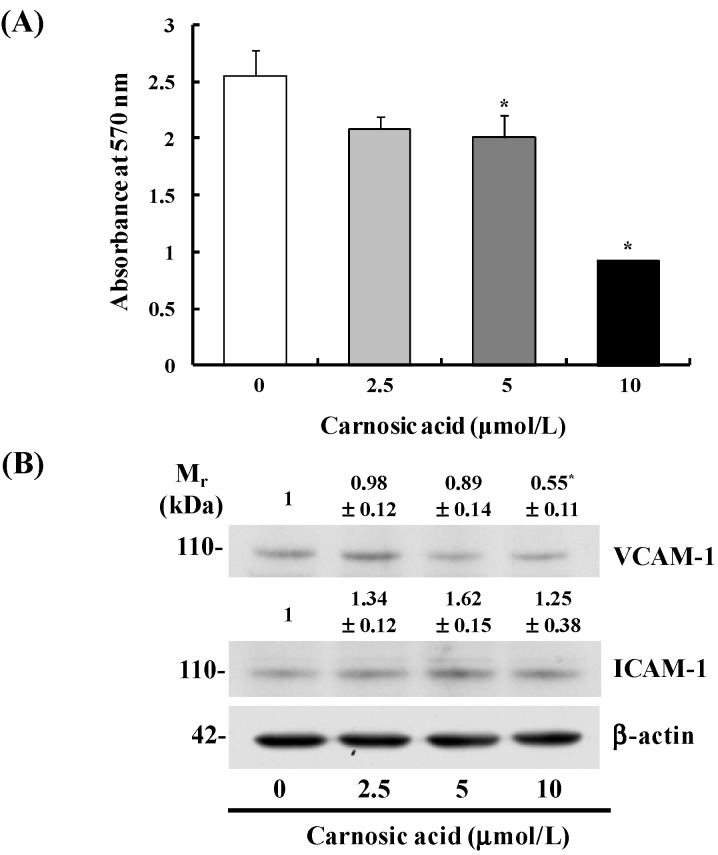
Carnosic acid inhibits adhesion of B16F10 cells. (**A**) Serum-starved B16F10 cells (5.0 × 10^4^ cells/well) were plated in CytoMatrix human collagen I cell adhesion strips, and incubated for 45 min in MEM containing 0–10 μmol/L carnosic acid. The cells were stained with 0.2% crystal violet, and the cell-bound stains were quantified colorimetrically. Each bar represents mean ± SEM (*n* = 6); (**B**) B16F10 cells (1.0 × 10^6^ cells/dish) were plated then serum-starved. Serum-starved cells were treated with carnosic acid for 12 h. Total cell lysates were then subjected to immunoblotting with an antibody raised against intercellular adhesion molecule (ICAM)-1 or vascular cell adhesion molecule (VCAM)-1. Photographs of chemiluminescent detection of the blots, which are representative of three independent experiments, are shown. The relative abundance of each band was estimated by densitometric scanning of the exposed films, and the expression levels were normalized to β-actin. The adjusted mean ± SEM (*n* = 3) of each band is shown above each blot. * Significantly different from the control (0 μmol/L carnosic acid), *p* < 0.05.

Carnosic acid significantly inhibited B16F10 cell adhesion to collagen type I in a dose-dependent manner (Figure 2A). Additionally, Western blot analyses of total cell lysates revealed that the levels of vascular cell adhesion protein (VCAM)-1 decreased by treatment with 10 μmol/L carnosic acid. However, the levels of intercellular adhesion molecule (ICAM)-1 were not affected significantly by carnosic acid treatment (Figure 2B).

### 2.4. Carnosic Acid Suppresses the EMT in B16F10 Cells

**Figure 3:**
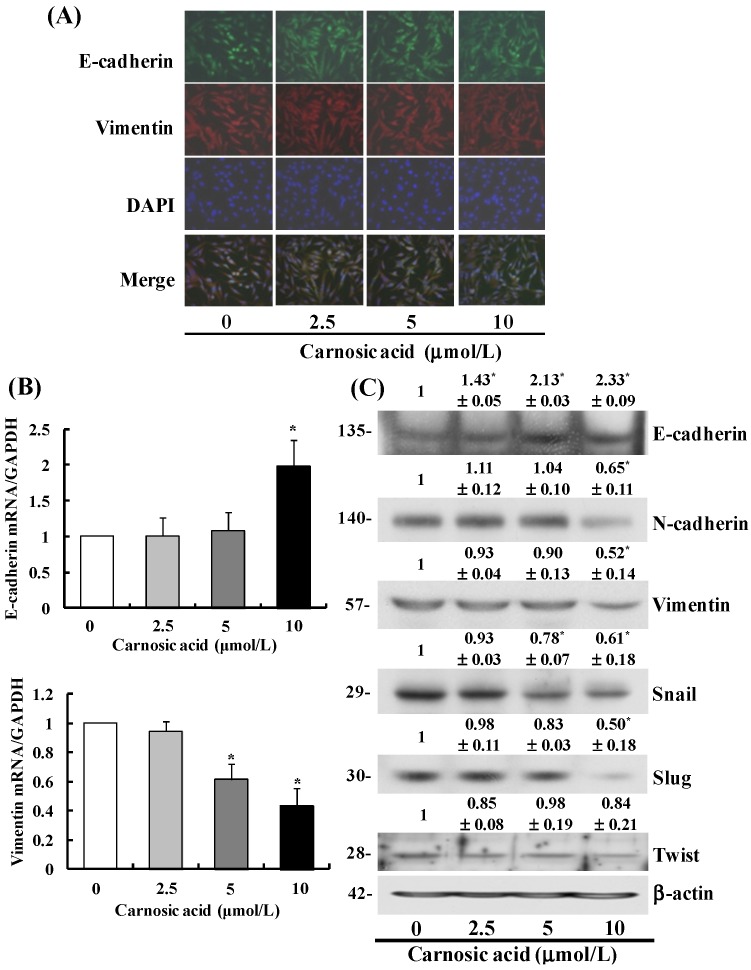
Carnosic acid blocks the epithelial-mesenchymal transition (EMT) in B16F10 cells. (**A**) Cells (5.0 × 10^4^ cells/well) were plated, treated with 0–10 μmol/L carnosic acid, fixed and permeabilized. The cells were incubated with an antibody to E-cadherin overnight at 4 °C, followed by extensive washing with PBS and incubation with Alexa Fluor 488 (green) for 1 h at room temperature. Anti-vimentin antibody was then added followed by overnight incubation at 4 °C. The cells were then washed with PBS and stained with Alexa Fluor 594 (red) for 1 h at room temperature. Nuclei were counterstained with DAPI (blue); (**B**) Serum-starved cells were incubated with carnosic acid for 6 h. Total RNA was isolated and reverse-transcribed, and real-time polymerase chain reaction was conducted. The levels of mRNA were normalized with those of GAPDH. Each bar represents mean ± SEM from three independent experiments; (**C**) Expression of the EMT markers was estimated by Western blot analysis. Photographs of chemiluminescent detection of the blots, which are representative of three independent experiments, are shown. The relative abundance of each band was estimated by densitometric scanning of the exposed films, and the expression levels were normalized to β-actin. The adjusted mean ± SEM (*n* = 3) of each band is shown above each blot. * Significantly different from the control (0 μmol/L carnosic acid), *p* < 0.05.

To determine whether carnosic acid induces the EMT in B16F10 cells, we identified changes in the expression of proteins involved in regulation of the EMT. Immunocytochemistry results revealed that carnosic acid increased E-cadherin expression, which is an epithelial phenotype marker [19], and suppressed that of the mesenchymal phenotype marker vimentin (Figure 3A). Reverse transcription-polymerase chain reaction (RT–PCR) results revealed that E-cadherin mRNA expression increased significantly, whereas that of vimentin decreased significantly in cells treated with carnosic acid (Figure 3B). Moreover, the results of Western blot analysis indicated that carnosic acid increased E-cadherin protein expression and decreased that of the vimentin and *N*-cadherin proteins (Figure 3C). To examine whether carnosic acid suppressed the expression of transcription factors involved in regulation of the EMT, the expression levels of snail, slug, and Twist were measured using Western blotting. Carnosic acid significantly decreased expression of the snail and slug proteins in B16F10 cells in a dose-dependent manner. However, Twist expression did not change in carnosic acid-treated cells (Figure 3C).

### 2.5. Carnosic Acid Inhibits AKT and Src Phosphorylation

**Figure 4:**
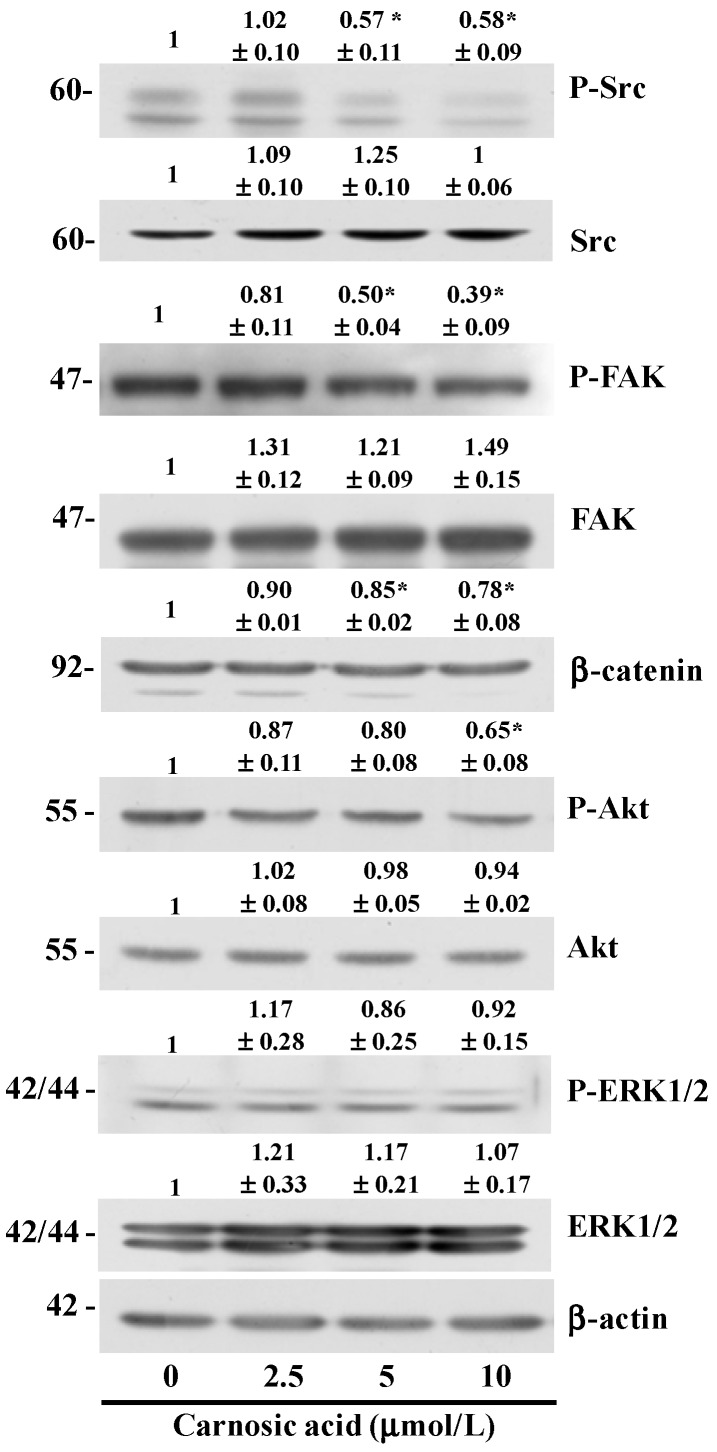
Carnosic acid inhibits phosphorylation of Akt, Src, and FAK**.** B16F10 cells (1.0 × 10^6^ cells/dish) were plated then serum-starved. Serum-starved cells were treated with carnosic acid for 6 h. Total cell lysates were then subjected to immunoblotting with the indicated antibodies. Photographs of chemiluminescent detection of the blots, which are representative of three independent experiments, are shown. The relative abundance of each band was estimated by densitometric scanning of the exposed films, and expression levels were normalized to β-actin (for Src, FAK, β-catenin, Akt, and ERK1/2) or the corresponding total protein (for P-Src, P-FAK, P-Akt, and P-ERK1/2). * Significantly different from the control (0 μmol/L carnosic acid), *P* < 0.05.

Several oncogenic pathways (peptide growth factors, Src, Ras, Ets, integrin, Wnt/b-catenin and Notch) regulate the EMT [20]. Src/FAK signaling is considered to be a mediator of cross-talk between cadherin- and integrin-mediated adhesion [21]. Carnosic acid reduced the ratio of P-Src/Src at 5 μmol/L. P-FAK/FAK ratio decreased in cells treated with 5 μmol/L carnosic acid. The levels of β-catenin also decreased significantly in cells treated with 5 μmol/L carnosic acid. Activation of the phosphatidylinositol kinase (PI3K)/AKT axis is also a central feature of the EMT [20]. Results of Western blot analyses revealed that treating B16F10 cells with 10 μmol/L carnosic acid for 6 h resulted in a decrease in AKT phosphorylation (Figure 4). These results indicate that carnosic acid inhibits the activation of AKT and Src/FAK, which may have contributed to the inhibition of EMT in the B16F10 cells.

## 3. Discussion

Rosemary (*Rosmarinus officinalis*) is commonly used as an herb, flavoring agent, and antioxidant in foods. The antioxidant properties of rosemary leave extracts are due to their phenolic diterpene contents such as carnosol and carnosic acid [22]. Carnosic acid exhibits antioxidant [23], anti-inflammatory [11], and anti-carcinogenic activities [14,15]. The ability of carnosic acid to inhibit tumor cell proliferation was reported earlier. For example, carnosic acid inhibits the proliferation of ER-negative breast cancer cells [12] and human HL-60 and U937 leukemia cells [13]. It has also been reported that 10–80 μg/mL *R. officinalis* extract, containing 31.7% carnosic acid, reduces the growth of M14 and A375 human melanoma cells [24]. Carnosic acid at higher concentrations (0–96 μmol/L) also decreases viability and inhibits migration and invasion of Caco-2 human cancer cells [15]. A decrease in viability can lead to a decrease in invasion and migration.

Our study was designed to investigate the anti-metastatic effects of carnosic acid and their underlying mechanisms with special focus on the EMT and migration of B16F10 mouse melanoma cells. We found that carnosic acid inhibited migration and adhesion in B16F10 melanoma cells at concentrations of 2.5–10 μmol/L without affecting cell viability (Figure 1B and Figure 2A). We also demonstrated that treating B16F10 cells with these concentrations of carnosic acid resulted in (1) decreased expression of MMP-9, TIMP-1, and uPA and increased TIMP-2 expression; (2) decreased expression of VCAM-1; (3) decreased expression of vimentin, snail, and slug and increased expression of E-cadherin; and (4) decreased phosphorylation of Akt, FAK, and Src. These results indicate that carnosic acid suppresses the EMT in melanoma cells as well as the migration and adhesion of melanoma cells. Further studies are needed to examine whether carnosic acid has anti-metastatic activity in animal cancer models.

Metastasis of tumor cells from the primary tumor to distant locations involves multiple distinct steps: migration/invasion through the ECM/basement membrane and entry into the lymphatic or circulatory system (intravasation); survival without contact with the ECM (resistance to anoikis); adhesion to the wall of lymphatic or blood vessels, and exit from the vessels (extravasation); and survival and proliferation in target tissues [25]. Anoikis is a form of apoptosis which is induced by inadequate or inappropriate cell–ECM interactions [26]. Cancer cells acquire the ability to resist anoikis, thereby surviving after detachment from their primary site [25]. Carnosic acid may stimulate anoikis, thereby inhibiting cell metastasis. Therefore, in this study, B16F10 migration was examined with the addition of carnosic acid at concentrations which do not inhibit cell growth in order to eliminate the possible effects of carnosic acid on apoptosis and/or anoikis. Future studies are needed to determine whether carnosic acid stimulates anoikis in B16F10 cells.

Migration and adhesion of cancer cells are complicated processes involving several proteolytic enzymes that participate in the degradation of environmental barriers such as the ECM and basement membrane [27]. MMPs, the largest group of zinc-dependent proteinases, are the most potent ECM degrading enzymes [28,29]. Among these proteases, the levels of MMP-2 and MMP-9 are high in various malignant tumors and are closely associated with the ability of cancer cells to invade and metastasize [30]. uPA is a serine protease involved in tissue remodeling and cell migration. uPA specifically cleaves the proenzyme/zymogen plasminogen to form the active enzyme plasmin, which then activates pro-MMP enzymes. Both uPA and the uPA receptor (uPAR) are overexpressed in various tumors with increased uPA levels observed mainly at the invasive margins of a tumor. It has also been shown that increased expression of uPA and uPAR in tumors and/or stromal cells is associated with the invasiveness of various tumors [31]. Therefore, MMP or uPA protein expression and their regulatory pathways are considered promising targets for chemopreventive agents and anticancer drugs [32]. In this study, we demonstrated that carnosic acid markedly decreased MMP-9 and uPA expression levels and increased TIMP-2 expression in B16F10 cells (Figure 1), which may be an important mechanism by which carnosic acid inhibits B16F10 cell migration and invasion.

TIMPs bind to MMPs and are specific endogenous inhibitors of MMPs [33]. In this study, we observed that carnosic acid inhibited TIMP-1 secretion and increased TIMP-2 secretion (Figure 1). High serum TIMP-1 levels have been associated with adverse prognoses in patients with endometrial carcinoma [34]. Our previous studies consistently showed that several anti-metastatic food components decrease secretion of TIMP-1 in several tumor cell types [35,36,37]. Consequently, these findings are reflective of a more complex role for TIMP-1 in tumor metastasis rather than simply the regulation of MMP activity. Taken together, the present results indicate that downregulation of MMP-9 and uPA and upregulation of TIMP-2 contribute to inhibit migration of B16F10 cells treated with carnosic acid.

The cyclic nucleotides (cAMP and cGMP) play a key role in signal transduction and the regulation of physiologic responses. Their intracellular levels are controlled by the large family of cyclic nucleotide phosphodiesterase (PDE)s [38]. The dysregulation of cAMP and/or cGMP generation by overexpression of PDE isoforms has been noted in several disease pathologies, including cancer and inflammation. For example, it has been reported that the levels of PDE activity are increased, affecting the ratio of cAMP/cGMP in an array of tumors [39]. PDE3 has been suggested to play a key role in cancer cell invasion and cell motility. Cilostazol, a specific PDE3 inhibitor, was reported to suppress human colon cancer cell motility [40]. In addition, *N*-(3,5-dichloropyrid-4-yl)-3-Cyclopentyloxy-4-methoxybenzamide, a selective PDE4 inhibitor, inhibits MMP-9 activity in the bronchoalveolar lavage fluids in ovalbumin-sensitized mice [41]. Recently, it has been reported that dibutyryl-cAMP inhibits HeLa human cervical cancer cell migration [42]. Our results showed carnosic acid to inhibit Akt phosphorylation (Figure 4) and MMP-9 secretion (Figure 1E). Akt phosphorylates PDE3B on serine-273 [43]. Therefore, it is possible that carnosic acid may be capable of decreasing the expression or activity of PDE3 and/or PDE4, thereby increasing cAMP levels, which, in turn, would inhibit B16F10 cell migration. Future *in vivo* and *in vitro* studies are warranted to determine whether carnosic acid inhibits the expression of PDEs and/or increases cAMP expression in cancer cells.

In addition to decreases in the migration of B16F10 cells, we also noted that carnosic acid inhibited adhesion of B16F10 cells and reduced the levels of the adhesion molecule VCAM-1 (Figure 2). Among the many changes in gene expression and protein function that occur during tumor progression, alterations in cell–cell and cell–matrix adhesion appear to play a crucial role promoting tumor cell migration, invasion, and metastatic propagation [44]. Our results show that carnosic acid inhibited cell adhesion, which is associated with a reduction in VCAM-1expression.

The EMT is believed to be a key mechanism in cancer progression whereby cancer cells gain more aggressive behavior. Many studies on various cancer tissues have demonstrated dislocation and downregulation of epithelial markers including E-cadherin, plakoglobin, and cytokeratin; upregulation of mesenchymal markers such as *N*-cadherin and vimentin, and expression of the EMT transcriptional drivers, snail1/2 and Twist [19,45]. Loss of E-cadherin expression is considered a hallmark event of the EMT, because reduced E-cadherin levels induce disruption of the epithelial cell-cell contacts that initiate a series of signaling events and a major cytoskeletal reorganization [45,46]. Expression of E-cadherin is negatively regulated by several zinc-finger transcription factors, including snail1, snail2, Twist, and ZEB1/ZEB2, each of which binds to E-boxes of the E-cadherin promoter and represses its transcription [47,48]. Our results demonstrate that carnosic acid downregulated the mesenchymal markers vimentin and *N*-cadherin and upregulated the epithelial marker E-cadherin (Figure 3), indicating that carnosic acid inhibits the EMT in B16F10 melanoma cells. Additionally, carnosic acid decreased the expression of snail and slug, whereas it did not significantly affect twist expression (Figure 3). Taken together, these results indicate that carnosic acid can inhibit the EMT in B16F10 cells, which is involved in inhibiting the expression of several transcription factors including snail and slug, which, in turn, results in an increase in E-cadherin expression.

A variety of cellular growth factors and signaling pathways induce the EMT, and the PI3K/Akt pathway is instrumental in the EMT. The EMT induced by activated Akt involves changes in the production or distribution of specific proteins, morphological changes, loss of apico-basolateral cell polarization, induction of cell motility, decreased cell–matrix adhesion, and decreased cell–cell adhesion [49]. AKT downregulates E-cadherin expression and promotes the EMT-like transition and invasiveness in carcinoma cells by inducing snail [50]. Akt also induces the production of metalloproteinases and cell invasion [51,52]. MMP-9 cooperates with snail to induce the EMT [53]. Our results show that carnosic acid inhibited Akt phosphorylation (Figure 4) and MMP-9 secretion (Figure 1E) and increased E-cadherin expression (Figure 3C). These results indicate that inhibiting AKT activation results in decreased snail and MMP expression, thereby leading to increased E-cadherin expression in carnosic acid-treated cells.

FAK and Src represent key players in the regulation of the cell–matrix interaction and in the formation of focal contacts, and mediate a variety of cell functions, such as the EMT, migration, invasiveness, and survival [21,54,55]. FAK is a major binding partner of Src, and the Src signaling pathway is an important component of adhesion changes associated with the EMT in carcinoma [56]. FAK may regulate cell invasion and migration by changing peripheral actin structures and focal adhesion, as well as MMP-mediated ECM degradation [57]. In the present study, carnosic acid inhibited Src and FAK phosphorylation (Figure 4) and reduced VCAM-1 (Figure 2) and MMP-9 expression (Figure 1). These results suggest that inhibiting the Src/FAK pathways as well as reducing expression of VCAM-1 and MMP-9 contributed to inhibit the EMT in carnosic acid-treated cells. *In vivo* studies are needed to determine whether carnosic acid inhibits the EMT, thereby inhibiting melanoma metastasis in animal tumor models and whether inhibition of the Src/FAK pathway directly contributes to inhibit the EMT.

## 4. Experimental

### 4.1. Materials

Carnosic acid was obtained from Sigma (St. Louis, MO, USA). Antibodies against uPAR, TIMP-1, TIMP-2, ICAM, VCAM-1, MMP-2, MMP-9, and PAI-1 were obtained from Santa Cruz Biotechnology (Santa Cruz, CA, USA). Antibodies against Akt, P-Akt, ERK1/2, P-ERK1/2, snail, slug, E-cadherin, *N*-cadherin, vimentin, and β-catenin were obtained from Cell Signaling Technology (Richmond, CA, USA). Anti-uPA antibody was obtained from Calbiochem (Darmstadt, Germany). An adhesion assay kit was obtained from Chemicon International (Temecula, CA, USA). Transwell filters were obtained from Costar (Corning, NY, USA). All other materials were obtained from Sigma.

### 4.2. Cell Culture

B16F10 melanoma, LLC lung cancer, and CT26 colon cancer cells were purchased from the American Type Culture Collection (Rockville, MD, USA). B16F10 cells were maintained in Minimum Essential Medium (MEM, Welgene, Daegu, Korea) with 100 mL/L fetal bovine serum (FBS) and 292 mg/L l-glutamine (Lonza, Walkersville, MD, USA). LLC and CT26 cells were maintained in Dulbecco’s Modified Eagle’s Medium and RPMI 1640 (Welgene), respectively, supplemented with 100 mL/L FBS. All maintenance media contained 100,000 U/L penicillin and 100 mg/L streptomycin.

### 4.3. Migration and Adhesion Assays

Cells for the Transwell migration assay were serum-starved or serum-deprived for 12 h with the following media: MEM (B16F10); DMEM + 10 mL/L FBS (LLC); and RPMI 1640 + 10 mL/L FBS (CT26). After serum-starvation or deprivation, the cells were plated onto Transwell filters in a 24-well plate. The Transwell filter was precoated with 10 μg type IV collagen. The lower chambers of the wells were filled with MEM + 100 mL/L FBS (B16F10), DMEM + 10 mL/L FBS + 1 g/L bovine serum albumin (BSA) (LLC) or RPMI 1640 + 10 mL/L FBS + 1 g/L BSA (CT26). FBS and BSA were used as chemoattractants. The cells were incubated with 0–10 μmol/L carnosic acid, and the migrated cells were stained with hematoxylin and eosin.

B16F10 cells were plated in human collagen type I-coated CytoMatrix Cell Adhesion Strips for the adhesion assay. The cells were incubated for 45 min in MEM with 0–10 μmol/L carnosic acid. The strips were rinsed three times with phosphate buffered saline (PBS) containing Ca^2+^/Mg^2+^ and stained for 5 min with 3 mg/L crystal violet in 100 mL/L ethanol. The cell-bound stains were then quantified by determining absorbance at 570 nm.

### 4.4. Western Blot Analyses

After treating the B16F10 cells for the indicated time periods with various concentrations of carnosic acid, the cells were harvested, washed with cold PBS, and lysed in the lysis buffer as detailed previously [58]. Conditioned media were collected and concentrated by centrifugal ultrafiltration using Amicon Ultra Centrifugal Filter Units (Millipore Ireland Ltd., Tullagreen, Carrigtwohill, Ireland). Protein contents were determined using a bicinchoninic acid protein assay kit (Thermo Scientific, Rockford, IL, USA). Proteins in total cell lysates (50 μg protein) and concentrated conditioned media (50 or 80 μg protein) were analyzed by Western blotting [36]. The relative abundance of each band was quantified using the Bio-profile Bio-1D application (Vilber-Lourmat, Marine La Vallee, France).

### 4.5. Real Time RT-PCR

Total RNA was extracted from the cells using the RNeasy Plus Mini kit (Qiagen, Valencia, CA, USA) based on the manufacturer’s protocol and reverse transcribed using SuperScript II reverse transcriptase (Invitrogen, Carlsbad, CA, USA) to synthesize complementary DNA, as described previously [35]. E-cadherin and vimentin mRNA was quantified by RT-PCR using a Rotergene 3000 PCR apparatus (Corbett Research, Sydney, Australia). Glyceraldehyde-3-phosphate dehydrogenase (GAPDH) was used as the endogenous expression standard. Real time PCR was performed using CYBER Premix with forward and reverse primers for E-cadherin, vimentin, and GAPDH [59]. PCR results were analyzed using Roter-gene software ver. 6 (Hilden, Germany).

### 4.6. Immunocytochemistry

Cells were plated on four-well chamber slides, allowed to attach, and then exposed to different concentrations of carnosic acid. The cells were fixed with 4% formaldehyde for 15 min, permeabilized with PBS containing 0.1% Triton X-100 for 30 min, and then blocked with 0.5% BSA in PBS for 1 h at room temperature. Cells to be double stained with antibodies were incubated with E-cadherin antibody at 4 °C overnight, followed by washing with PBS and incubating with secondary antibody for 1 h at room temperature. A vimentin antibody was then added followed by overnight incubation at 4 °C. Cells were then washed three times with PBS and stained with secondary antibody for 1 h at room temperature. Cell nuclei were stained with DAPI. Cells were mounted, and observed under an optical microscope system (Axiomager, Carl Zeiss, Jena, Germany).

### 4.7. Statistical Analysis

Each experiment was repeated at least three times, and the results are presented as the means ± SEM. Differences between the treatment groups were assessed via Duncan’s multiple range test (SAS system for Windows v. 9.2; SAS Institute, Cary, NC, USA). Differences were considered significant at *p* < 0.05.

## 5. Conclusions

In summary, we demonstrated that the biological functions and activities of carnosic acid are sufficient to block migration of B16F10 melanoma cells. Carnosic acid inhibited the migration and adhesion of melanoma cells in a dose-dependent manner. Carnosic acid increased expression of E-cadherin and reduced expression of vimentin and *N*-cadherin. The downregulation of MMP-9, uPA, and VCAM-1 expression and upregulation of TIMP-2 contributed to inhibit migration and adhesion in carnosic acid-treated cells. Downregulation of slug and snail expression lead to a reduction in the EMT in B16F10 melanoma cells. Inhibiting Akt, Src, and FAK activation was also responsible, at least in part, for inhibiting cell migration and the EMT in carnosic acid-treated cells. These results provide a molecular basis for future *in vivo* studies to examine whether carnosic acid inhibits the EMT thereby leading to suppression of melanoma metastasis.
